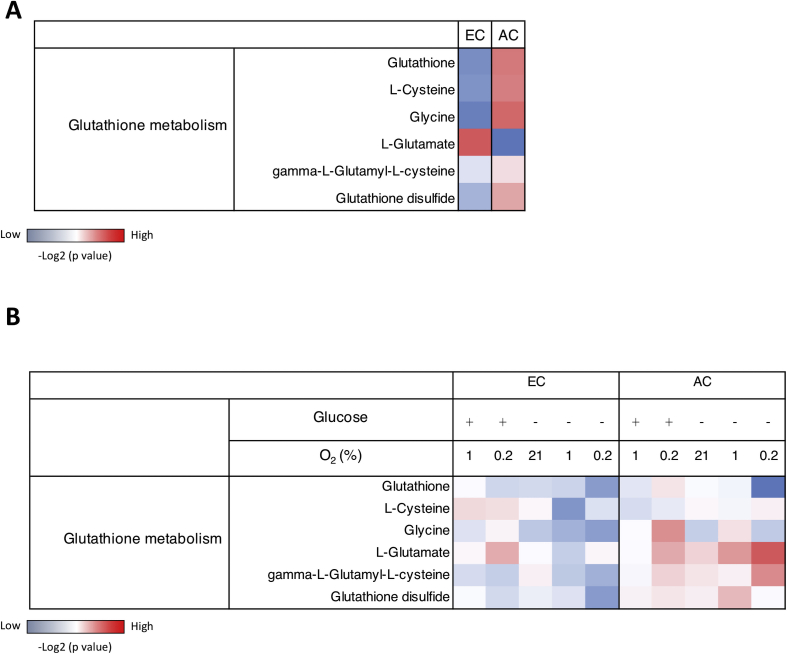# Astrocyte glutathione maintains endothelial barrier stability

**DOI:** 10.1016/j.redox.2020.101576

**Published:** 2020-05-19

**Authors:** Sheng-Fu Huang, Alaa Othman, Alexey Koshkin, Sabrina Fischer, David Fischer, Nicola Zamboni, Katsuhiko Ono, Tomohiro Sawa, Omolara O. Ogunshola

**Affiliations:** a Institute for Veterinary Physiology, University of Zurich, Winterthurerstrasse 260, CH-8057, Zurich, Switzerland; b Zurich Center for Integrative Human Physiology, University of Zurich, Winterthurerstrasse 190, CH-8057, Zurich, Switzerland; c Department of Biology, Institute of Molecular Systems Biology, Eidgenössische Technische Hochschule Zürich, Otto-Stern-Weg 3, CH-8093, Zurich, Switzerland; d Institute of Zoology, University of Basel, Vesalgasse 1, CH-4051, Basel, Switzerland; e Functional Genomics Center Zurich, University of Zurich, Winterthurerstrasse 260, CH-8057, Zurich, Switzerland; f Department of Microbiology, Graduate School of Medical Science, Kumamoto University, 1-1-1 Honjo, Kumamoto 860-8556, Japan

**Keywords:** Neurovascular unit, Metabolic communication, Barrier stability, Tight junction, Blood-brain barrier, Redox balance

## Abstract

Blood-brain barrier (BBB) impairment clearly accelerates brain disease progression. As ways to prevent injury-induced barrier dysfunction remain elusive, better understanding of how BBB cells interact and modulate barrier integrity is needed. Our metabolomic profiling study showed that cell-specific adaptation to injury correlates well with metabolic reprogramming at the BBB. In particular we noted that primary astrocytes (AC) contain comparatively high levels of glutathione (GSH)-related metabolites compared to primary endothelial cells (EC). Injury significantly disturbed redox balance in 10.13039/501100000780EC but not AC motivating us to assess 1) whether an AC-10.13039/501100000780EC GSH shuttle supports barrier stability and 2) the impact of GSH on 10.13039/501100000780EC function. Using an isotopic labeling/tracking approach combined with Time-of-Flight Mass Spectrometry (TOF-MS) we prove that AC constantly shuttle GSH to EC even under resting conditions - a flux accelerated by injury conditions *in vitro*. In correlation, co-culture studies revealed that blocking AC GSH generation and secretion via siRNA-mediated γ-glutamyl cysteine ligase (GCL) knockdown significantly compromises EC barrier integrity. Using different GSH donors, we further show that exogenous GSH supplementation improves barrier function by maintaining organization of tight junction proteins and preventing injury-induced tight junction phosphorylation. Thus the AC GSH shuttle is key for maintaining EC redox homeostasis and BBB stability suggesting GSH supplementation could improve recovery after brain injury.

## Highlights

• Astrocytes maintain better redox homeostasis during injury conditions than brain endothelial cells.
• Astrocyte-secreted glutathione abrogates injury-induced endothelial permeability.
• Exogenous GSH prevents injury-induced tight junction disruption.
• Better understanding of metabolic paracellular crosstalk could offer more opportunities to safeguard BBB integrity.

## Graphical abstract

**Figure:**
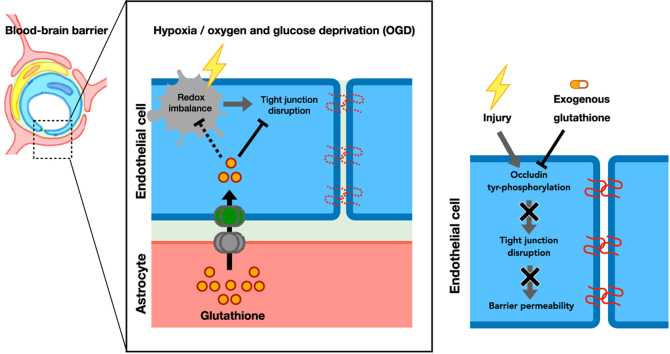

## 1 Significance

Improving brain vascular function to accelerate disease recovery remains an unmet medical need. Here we show that astrocyte (AC)-derived glutathione (GSH) plays a strategic role in endothelial (EC) stability by suppressing EC tight junction phosphorylation and delocalization. Importantly, blocking AC GSH shuttling disrupts the endothelial barrier resulting in increased permeability. Thus maintaining this transfer during injury scenarios is critical. Our data provides significant insight into (patho)physiological metabolic BBB regulation and suggests GSH treatment may be an effective way to combat EC dysfunction and improve vascular health.

## 2 Introduction

Vascular health underlies proper physiological function. Early blood-brain barrier (BBB) breakdown and/or dysfunction occurs in many neurological diseases, and significantly contributes to disease progression [[1], [2], [3]]. Multiple studies suggest that stabilizing brain vascular function in patients with neurological disease could arrest or even reverse the course of brain disorders [2], but ways to attain this goal remain elusive. Highly specialized endothelial cells (EC) that are in close contact with perivascular astrocytes and pericytes, form the inner wall of brain microvessels. The stabilization of EC tight junction complexes mediates barrier tightness and restricts the passage of substances to and from the bloodstream thus maintaining cerebral ion and metabolic balance [3,4]. We are convinced that better understanding of how perivascular cells regulate both physiological and pathological EC responses will provide valuable insight into how to modulate BBB functionality.

Astrocyte (AC) endfeet ensheath brain microvessels as well as connect to neurons thus functioning as regulators of different cellular compartments. Direct contact between AC and neurons allows exchange of multiple substances that sustain neuronal survival and function. An important example is the release of supportive metabolites such as glutathione (GSH) and glutamine. GSH is a critical antioxidant in the brain that maintains cellular oxidative homeostasis [5]. GSH metabolic crosstalk rapidly contributes to maintaining neuronal redox homeostasis [6], supporting neurotransmitter recycling [7,8] and regulating neuronal gene expression programs [9]. In contrast, little is known of how metabolites impact BBB integrity [4], but it seems logical that a similar AC-10.13039/501100000780EC crosstalk could also support barrier function.

We previously performed metabolomic profiling of primary AC and EC to obtain detailed insight into cell-specific regulation of critical pathways during resting and different injury conditions [10]. A number of metabolites and pathways that were reduced in EC but elevated or stabilized in AC during injury were identified. We considered that AC secretion of such metabolites, especially those known to facilitate cell survival or adaptation to adverse situations, could be key in supporting 10.13039/501100000780EC function. GSH was of particular interest due to its critical anti-oxidant role and the fact that GSH-deficiency has already been observed in many BBB impairment-associated brain diseases in patients [11,12]. GSH deficit was also shown to induce barrier leakage in a rat model [13] and its administration prevented endothelial oxidative imbalance *in vitro* [14,15].

We hypothesized that GSH is critical for brain vascular function and its secretion by AC promotes BBB stability during injury conditions i.e. when EC GSH generation is impeded. Using an isotopic labeling/tracking approach combined with Time-of-Flight Mass Spectrometry (TOF-MS) we prove that AC secreted GSH is constantly shuttled to EC, and this flux is accelerated by injury conditions. Next, we demonstrate that GSH synthesis-deficient AC (via siRNA-mediated γ-glutamyl cysteine ligase knockdown) lose their ability to protect the barrier under injury conditions. Finally, in the absence of AC, sole GSH supplementation abrogates injury-induced EC permeability. Taken together, better insight of metabolic shuttling and reprogramming may provide innovative ways to modulate BBB function.

## 3 Results

### 3.1 Changes in glutathione-related metabolites during injury conditions

**Fig. 1:**
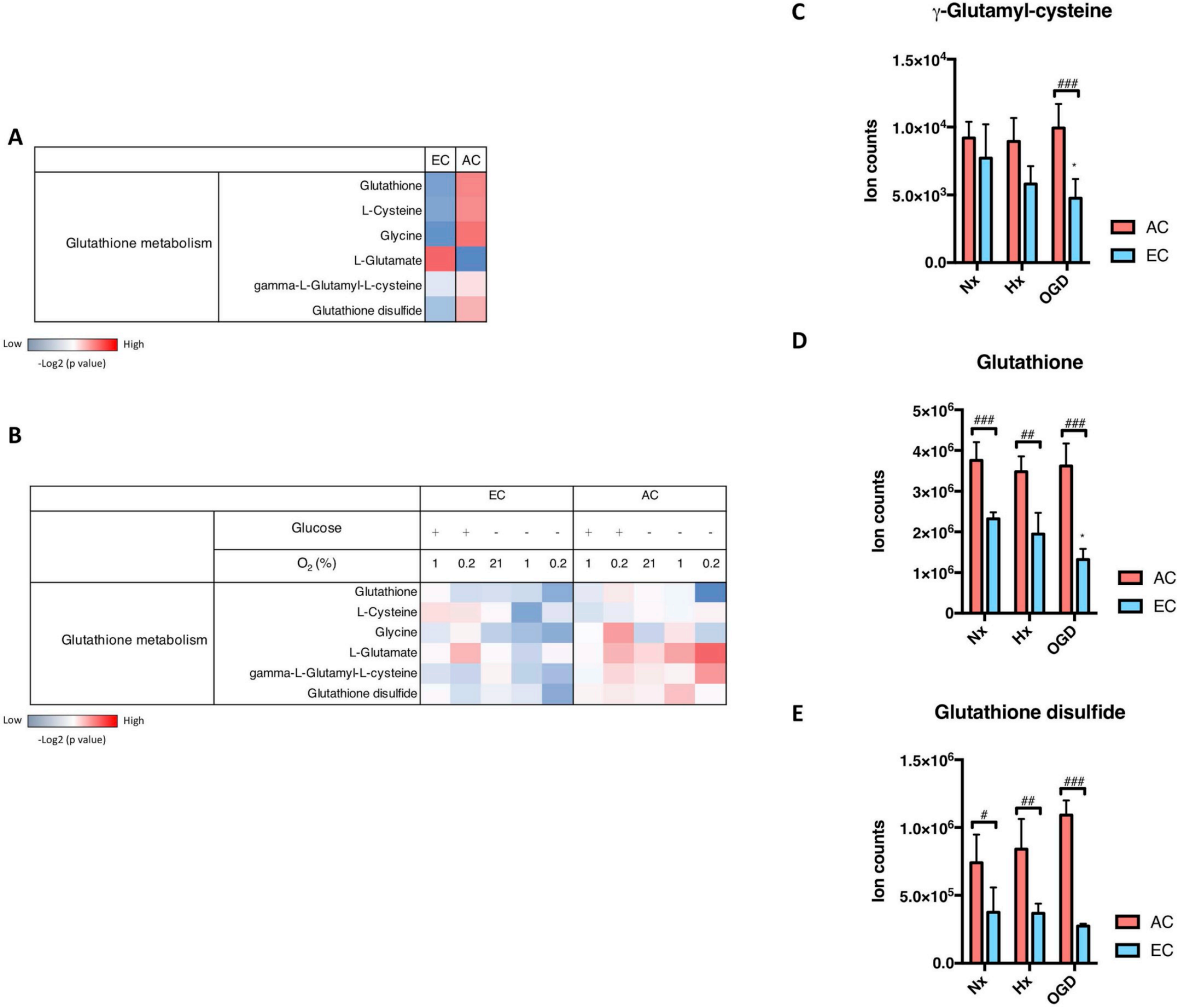
Changes in glutathione-related metabolites during injury conditions. Levels of metabolites that participate in glutathione (GSH) metabolism in rat brain primary astrocyte (AC) and endothelial cell (EC) were measured by LC-MS after exposure to oxygen deprivation (1% O_2_ and 0.2% O_2_) combined with or without glucose (Glc) withdrawal for 24h. ***(A)*** Heatmap shows the relative activity of the major GSH-related metabolites comparing the two cell types under normoxia (Nx). ***(B)*** Heatmap of GSH metabolic alterations during injury was generated by comparison with their Nx control with increased metabolite level in red and decreased metabolite level in blue. ***(C-E)*** Ion counts of three GSH metabolism indicators, g-glutamyl-cysteine ***(C)***, GSH ***(D***) and GSH disulfide ***(E)***, during injury is presented separately. Hypoxia (Hx, 1% O_2_ with glucose) and OGD (1% O_2_ without glucose). The metabolite intensities were normalized to total ion counts. The -Log2(P value) for each metabolite was calculated using unpaired T-test. *P<0.05; One-way ANOVA compared to Nx. ^#^P<0.05, ^##^P<0.01, ^###^P<0.001; Two-way ANOVA, compared to astrocytes (AC) in the same condition. Mean ± SD. n = 4.

In a previous study we used untargeted LC-MS to obtain comprehensive metabolomic profiles of primary rat brain microvascular endothelial cells (EC) and primary rat astrocytes (AC) exposed to different conditions (Huang et al. in press). Samples were collected and extracted after 24h normoxia (Nx), hypoxia (Hx) or near anoxia (Ax) in media with or without glucose (±Glc) to simulate ischemia *in vitro*. We noted that regulation of key metabolites of the GSH pathway was quite different in the two cell types. A comparison relative to the baseline (Nx) composition of each cell is shown in a heatmap with increased and decreased metabolites indicated in red and blue respectively (Fig. 1A). Major metabolites supporting GSH synthesis such as cysteine and glycine were clearly more abundant in AC (Fig. 1A). Furthermore, cellular GSH and GSH disulfide levels were overrepresented in AC compared to EC (Fig. 1A).

Next we investigated how injury modulates cellular GSH metabolism in AC and EC by comparing the injury profiles with Nx baseline conditions. Heat maps show oxygen and glucose deprivation stimulate AC GSH metabolic activity whereas a clear reduction occurs in EC (Fig. 1B). Interestingly, under the most severe injury condition (0.2% O_2_ without glucose) GSH and glycine were strongly reduced in AC, whereas γ-glutamyl-cysteine, a precursor that determines the GSH synthesis rate [16], remained high. This implies that AC maintain GSH metabolism even during severe conditions. In complete contrast, EC GSH metabolism was diminished under virtually all conditions and particularly in the absence of glucose. We directly compared levels of three GSH activity indicators in AC and EC during hypoxia (1% O_2_ with glucose) and oxygen and glucose deprivation (OGD) i.e. 1% O_2_ without glucose (Fig. 1C–E). At baseline both cells displayed similar amounts of the intermediate product γ-glutamyl-cysteine with an injury-induced reduction observed only in EC (Fig. 1C). Much higher levels of the end products GSH and GSH disulfide were consistently measured in AC (Fig. 1D and E) with EC GSH levels strongly reduced during OGD (Fig. 1D). Thus AC have comparatively better stores and greater capacity to generate/use GSH in injury situations.

### 3.2 Oxidative homeostasis is stable in AC but not EC

**Fig. 2:**
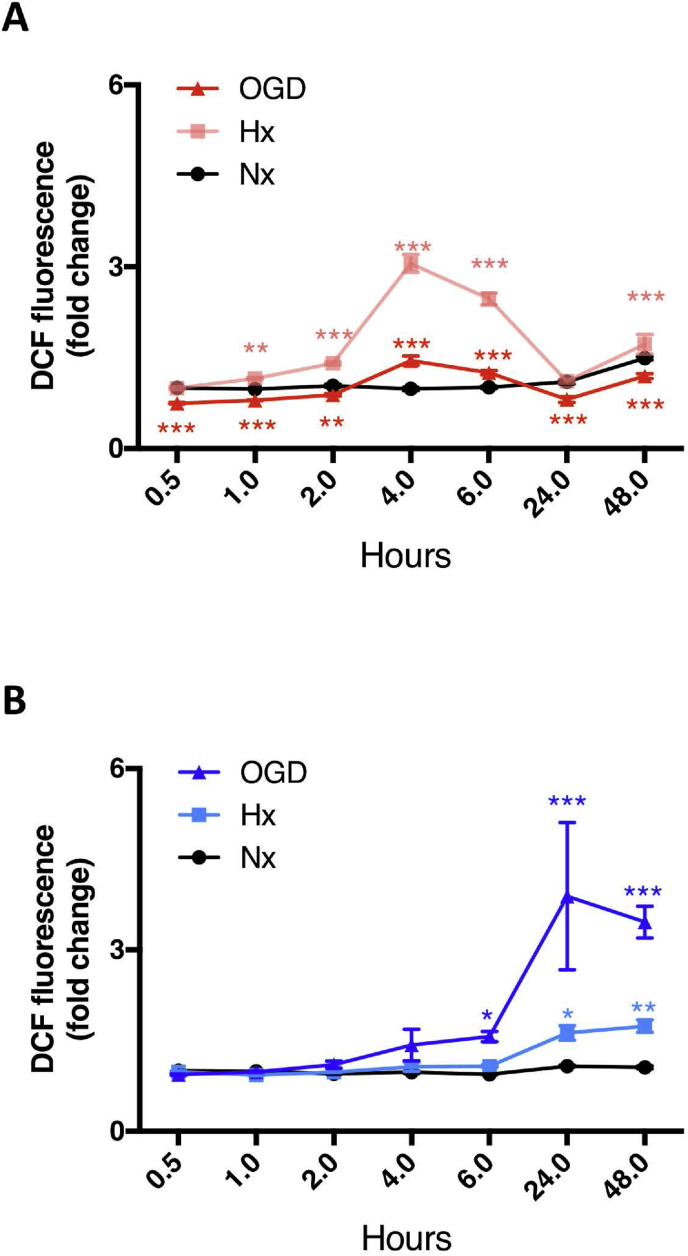
Oxidative homeostasis is stable in AC but not EC. **(A and B)** Oxidative stress measurements were performed in primary AC ***(A)*** and EC ***(B)*** exposed to hypoxia (Hx, 1% O_2_ with glucose) and OGD (1% O_2_ without glucose). Nx, 21% O_2_ with glucose group was used as control. *P < 0.05, **P < 0.01, ***P < 0.001; Two-way ANOVA compared to Nx at the same time point. Mean ± SD. n = 3.

GSH efficiently maintains cellular redox balance [5]. To know whether the redox equilibrium of AC and EC correlated with their GSH metabolic activity, we measured reactive oxygen species (ROS) levels for 48 h in hypoxic and ischemic cells. Within 4h hypoxia induced a 3-fold increase in AC ROS activity that returned to normoxic baseline by 48h (Fig. 2A). Intriguingly, the severest condition of oxygen and glucose withdrawal in AC had less dramatic effects on ROS levels and never reached those of hypoxic values (Fig. 2A). In contrast, ROS accumulation in EC (Fig. 2B) was insult severity-dependent with OGD inducing higher ROS levels than hypoxia. Notably OGD-induced ROS accumulation in EC began only after 6 h and remained consistently elevated by 2–4 fold at 48h compared to Nx baseline (Fig. 2B). These results highlight a cell-specific and time-dependent modulation of redox balance during injury conditions.

### 3.3 Injury increases GSH secretion by AC

**Fig. 3:**
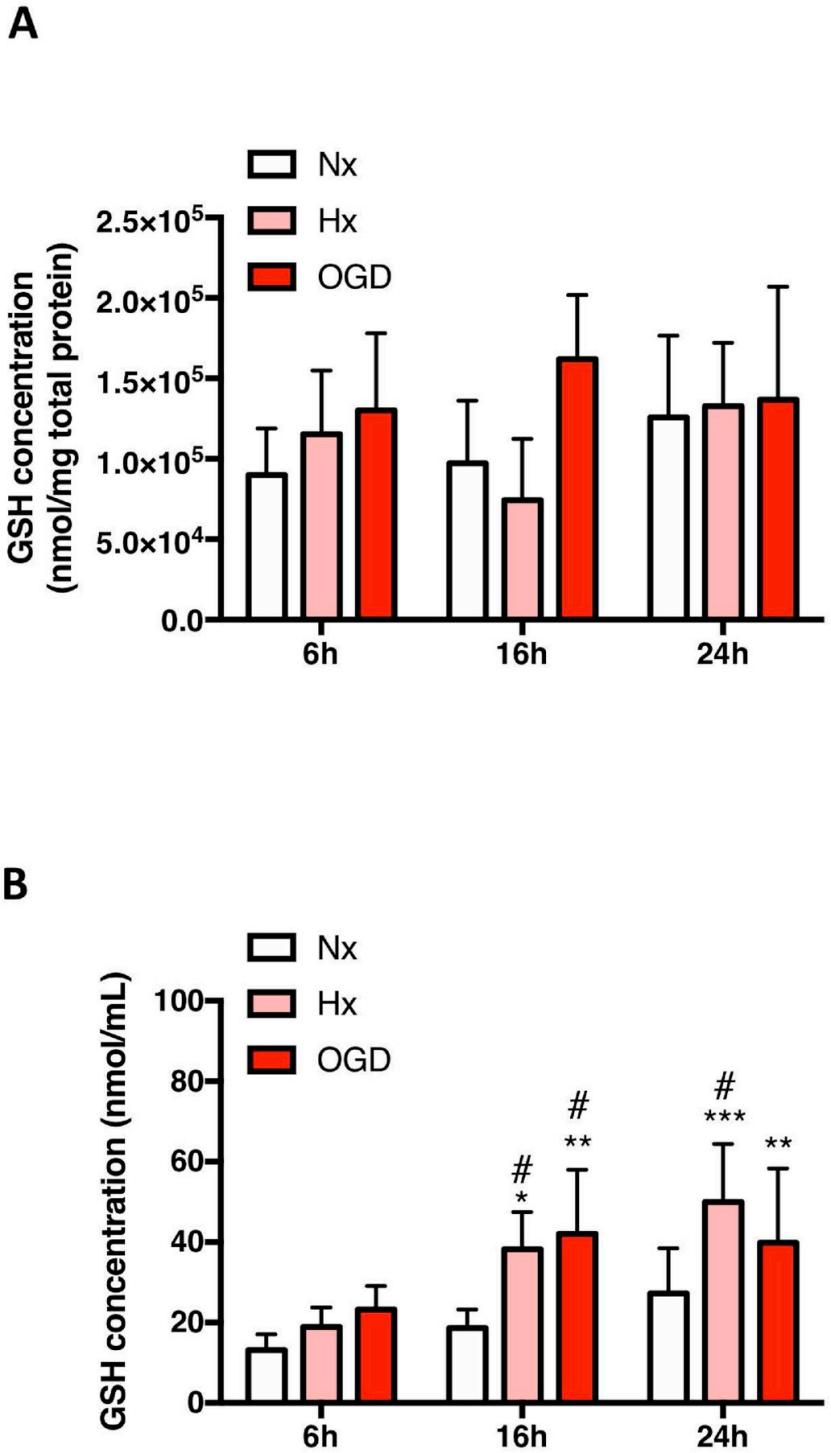
Injury increases GSH secretion by AC. ***(A and B)*** Changes in intracellular and extracellular GSH levels in primary AC after 6h, 16h and 24h Hx and OGD were measured in cell lysates ***(A)*** and culture media ***(B)*** respectively. Concentrations were normalized to either 1 mg total protein or 1 mL media to facilitate comparison between the different conditions and cell type. *P<0.05, **P<0.01, ***P<0.001; two-way ANOVA compared to Nx baseline. ^#^P<0.05; Two-way ANOVA, compared to injury 6h baseline. Mean ± SD. n=6.

Since secretion of GSH and its related metabolites by AC could benefit EC in addition to neurons *in vivo* we assessed AC intracellular and extracellular GSH levels during normoxia, hypoxia and OGD for up to 24h. Interestingly, endogenous GSH levels were consistently maintained at normoxic levels with OGD tending to increase the quantities (Fig. 3A). Extracellularly a time- and injury-dependent increase of more than two-fold was noted compared to normoxic conditions (Fig. 3B). Thus injury conditions stimulate GSH secretion but do not alter endogenous homeostasis consistent with GSH associated pathways being constantly active and modulated in AC during injury situations (Fig. 1B). To date, a GSH-specific exporter has not been identified but multidrug resistance proteins (MRP) are considered to be the major transporter family involved in GSH secretion [17]. In this regard injury-induced AC MRP2 mRNA (Supplementary information Fig. S1A) and protein expression was observed (Supplementary information Fig. S1B) whereas MRP1 and MRP4 expression was unchanged (data not shown). Gamma glutamyl transpeptidase (GGT) plays a key role in extracellular GSH catabolism and supporting intracellular oxidative stress homeostasis. We observed that messenger RNA levels of GGT were strongly induced during hypoxia and particularly OGD (Supplementary information Fig. S1C) but no change in protein levels was detected (Supplementary information Fig. S1D).

### 3.4 GSH is constantly shuttled from AC to EC

**Fig. 4:**
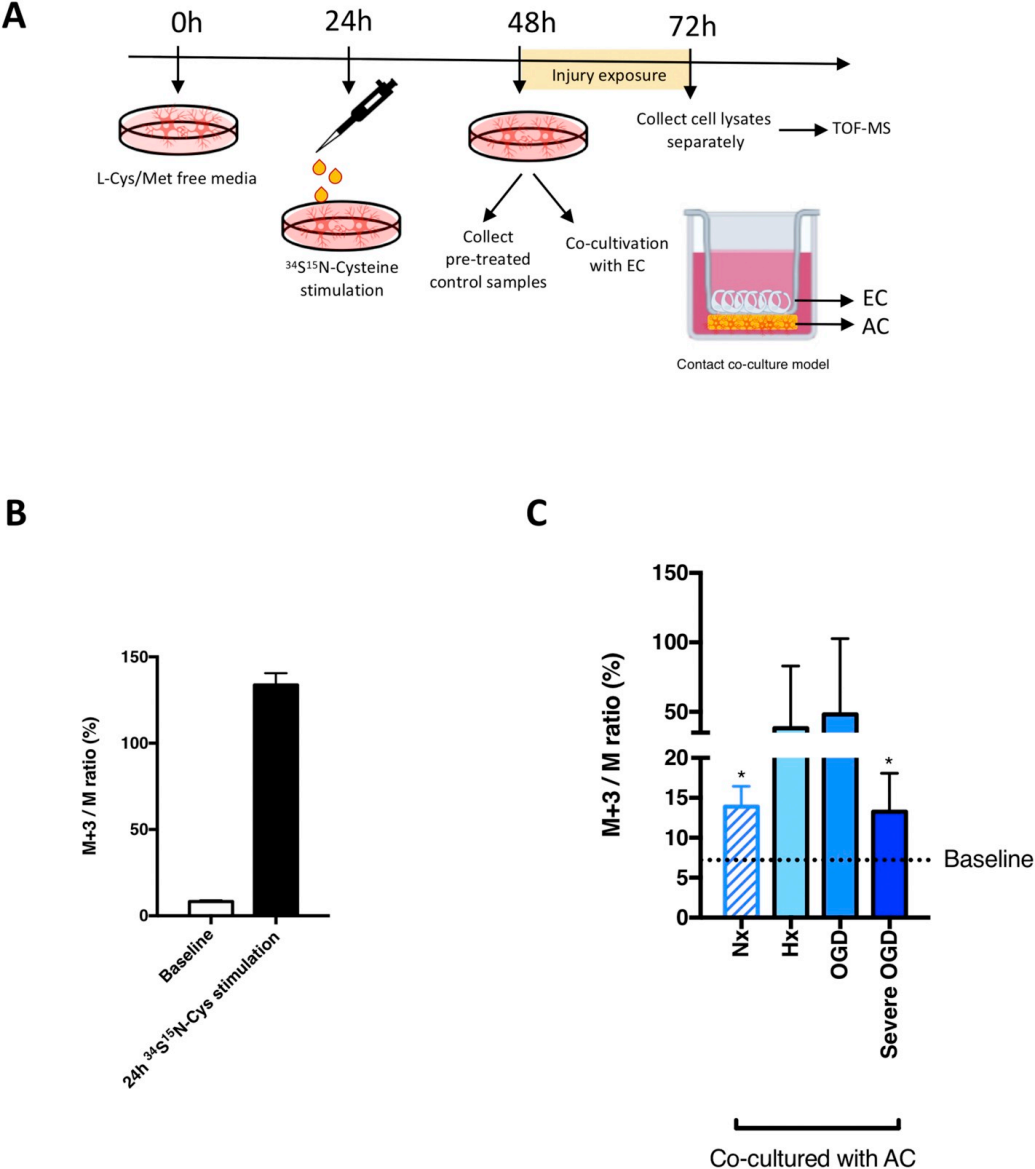
GSH is constantly shuttled from AC to EC. To investigate if EC take up AC-derived GSH, a stable isotope labelling approach was combined with Time-of-Flight Mass Spectrometry (TOF-MS) to specifically track AC synthesized GSH*.****(A)*** The schematic shows the experimental setup. Endogenous GSH was depleted by incubating with L-Cys/Met free media for 24h. ^34^S^15^N-cysteine was employed to stimulate AC endogenous ^34^S^15^N-GSH generation. Isotope-labeled AC were co-cultured with EC under different conditions for 24h then cell lysates were analyzed by TOF-MS. ***(B)*** Levels of intracellular ^34^S^15^N-GSH was measured in untreated AC lysate (baseline) and after 24h isotope stimulation*.****(C)*** After co-culture, levels of labeled GSH in EC lysates were measured. *P<0.05, **P<0.01, ***P<0.001; Student’s T- test compared to baseline. Mean ± SD. n=4.

To investigate if EC take up the GSH secreted by AC, a stable isotope labeling approach combined with TOF-MS was used to monitor its movement. As cysteine is an essential GSH building block, ^34^S^15^N-cysteine (Cys) isotope was incorporated into AC GSH molecules as schematically depicted (Fig. 4A). First, endogenous GSH levels were depleted by culturing AC in methionine/cysteine-free culture media for 24h. Subsequently the AC were incubated in ^34^S^15^N-Cys-containing media for 24h to boost biosynthesis of endogenously labeled ^34^S^15^N-GSH. The ratios of ion intensities of GSH (considering both mass accuracy and TOF resolution) were compared in AC treated with and without ^34^S^15^N-Cys. Using an annotation depicting the abundance of isotopes, M is natural GSH monoisotopic mass and M+3 is enriched metabolite which is 3 Da heavier than the monoisotopic GSH. The M+3/M ratio, corresponding to the natural isotopic abundance in the baseline samples, is expected to increase in samples treated with the isotope (due to the inclusion of labeled GSH in the cellular GSH pool). Accordingly, the natural abundance of labeled GSH (M+3/M) was 6–7% in cells not incubated in ^34^S^15^N-Cys and exceeded 135% after 24h stimulation confirming successful incorporation into the intracellular GSH pool (Fig. 4B). Next, to test our hypothesis that AC-derived GSH is shuttled to EC, we co-cultured labeled AC with EC in Transwells™ [18] prior to 24h injury exposure (Fig. 4A). Samples from the different compartments were then collected and analyzed to assess if transfer occurred. Interestingly, intracellular levels of labeled GSH in both mono- and co-cultured AC lysates always corresponded to baseline values (6%–8%), suggesting extended exposure results in metabolic dilution of the M+3 enrichment (Supplementary information Fig. S2). Unexpectedly, measurement of EC intracellular metabolites after normoxic (Nx) co-culture showed labeled GSH levels were increased by up to 2-fold compared to baseline within 24h. Thus metabolic shuttling exists in the absence of injury (Fig. 4C). Injury exposure further expedited enrichment of labeled GSH (Fig. 4C) demonstrating increased paracrine crosstalk. Notably, GSH shuttling occurs even during harshest conditions as levels similar to normoxia were maintained during severe OGD (Ax-Glc) (Fig. 4C). Thus GSH shuttling between AC and EC is constantly active.

### 3.5 Paracrine GSH shuttling is crucial for barrier maintenance during injury

**Fig. 5:**
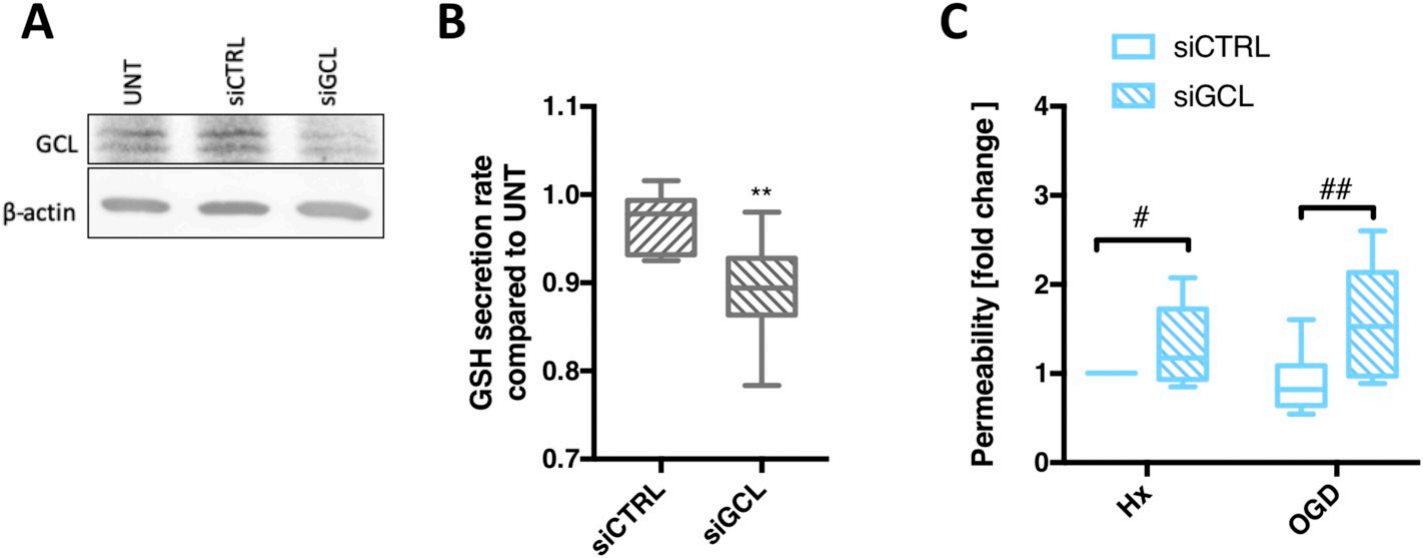
Paracrine GSH shuttling is crucial for barrier maintenance during injury. ***(A)*** AC were transfected with GCL small Interfering RNA (siGCL). A representative immunoblot after 48h transfection shows normoxic GCL expression levels. ***(B)*** The rate of GSH release by normoxic AC after GCL knockdown was measured post transfection at 24h and 48h. ***(C)*** Permeability of the EC barrier after co-culture with transfected AC under different conditions for 48h was measured by lucifer yellow flux. **P < 0.01, compared to Nx siCTRL. ^#^P < 0.05, ^##^P < 0.01; Student's T-test compared to injury control (siCTRL). n = 8. (For interpretation of the references to colour in this figure legend, the reader is referred to the Web version of this article.)

To prove GSH shuttling supports barrier function, we employed siRNA to silence γ-glutamyl cysteine ligase (GCL) gene and disrupt the rate limiting step of GSH synthesis [5]. Post siGCL transfection, protein levels were significantly reduced (Fig. 5A) and GSH secretion rate was successfully reduced by 10–20% compared to untreated controls (Fig. 5B). GSH-deficient AC were then co-cultured with primary EC on Transwells™ and exposed to hypoxic/OGD for 48h. Barrier function was measured by lucifer yellow flux. In agreement with their barrier supportive role [4,18], co-culture of EC with untreated (UNT) AC prevented injury-induced barrier leakage (Fig. S3). Remarkably, when co-cultured with GSH-deficient AC (siGCL) significantly increased paracellular flux was measured under both injury conditions compared to siCTRL (Fig. 5C). Thus impeding GSH shuttling compromises AC protective effects and directly impairs the ability of EC to maintain their barrier function. Clearly the shuttle reinforces barrier stability.

### 3.6 Boosting EC GSH levels prevents injury-induced BBB breakdown

**Fig. 6:**
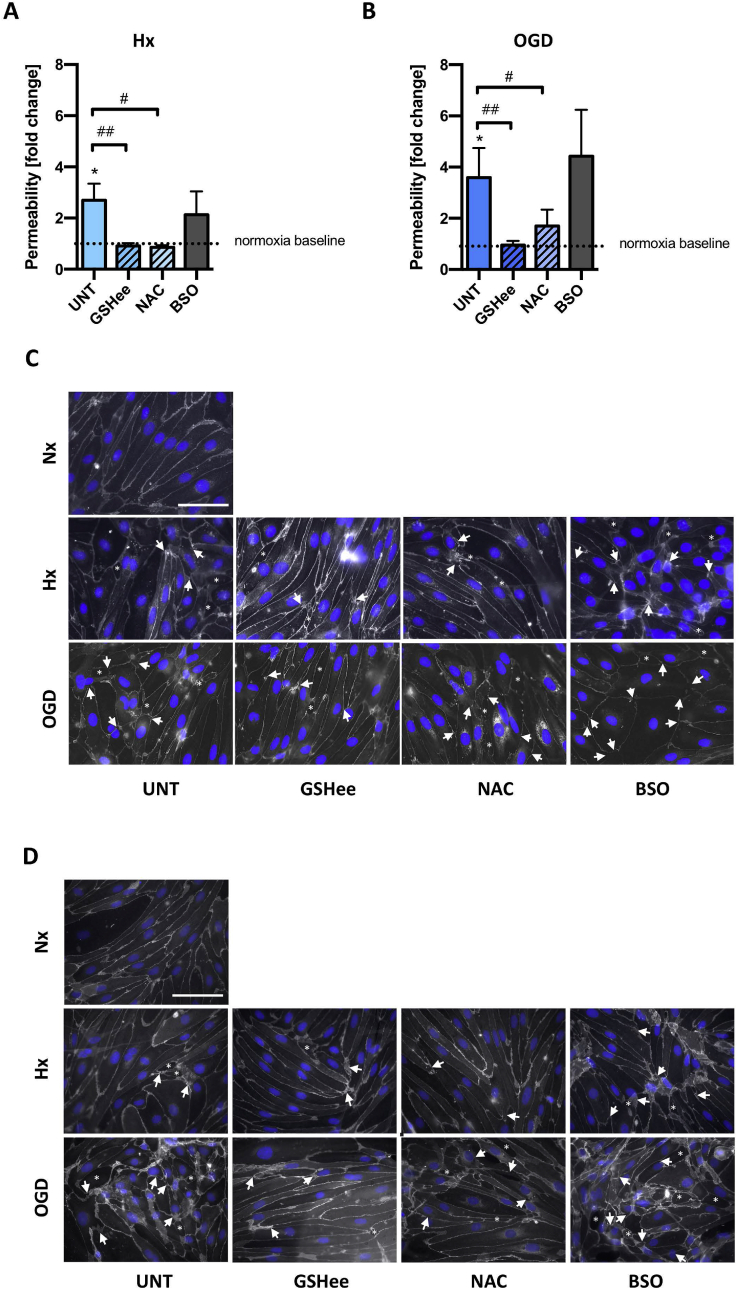
Boosting EC GSH levels prevents injury-induced BBB breakdown. Primary EC monolayers were either untreated (UNT) or exposed to GSH compounds and exposed to hypoxia/OGD for 48h. GSH enhancers (5mM NAC and 5mM GSHee) and GSH synthetase inhibitor (200mM BSO) were applied. ***(A and B)*** EC barrier leakage was measured under Hx and OGD using lucifer yellow. ***(C and D)*** Representative immunofluorescent images of claudin-5 and b-catenin localization in EC. Note the disconnection and delocalization of junctions in areas marked by arrows. Asterisks indicate inter-EC gap formation and holes. *P<0.05; One-way ANOVA compared to Nx baseline. ^#^P<0.05, ^##^P<0.01, ^###^P<0.001; Two-way ANOVA, compared to injury untreated (UNT). Mean ± SD. n = 5.

As EC GSH metabolism is increasingly shutdown during injury, we asked if barrier impairment can be prevented by providing the metabolite exogenously. Permeability of confluent primary EC monolayers treated with GSH compounds prior to hypoxic/ischemic exposure for 48h was measured. In untreated samples the degree of barrier permeability correlated with injury severity (Fig. 6A and B) as expected. Excitingly, enhancing GSH levels using GSHee and NAC prevented barrier leakage not only in hypoxia but also during OGD (Fig. 6A and B). Notably, GSHee maintained the BBB as tight as the normoxic controls in both injury conditions. The GSH synthesis inhibitor (BSO) showed no additional negative effect compared to controls (UNT, Fig. 6A and B).

Using immunostaining we next tested if exogenous GSH improved BBB stability by suppressing injury-induced tight and adherens junction delocalization. As expected, injury severity progressively disrupted junctional localization at cell-cell borders as observed by discontinuous and frayed organization of claudin-5 (arrows, Fig. 6C) and occludin (data not shown) in hypoxic cells compared to normoxic controls. Injury also disrupted β-catenin retention at cell-cell borders (arrows, Fig. 6D). Overall during injury the cells lost both morphology and close organization with gaps increasingly visualized (asterisks). Consistent with the functional data, GSH enhancers prevented tight and adherens junction disruption in both injury conditions although NAC was consistently less effective than GSHee (Fig. 6C and D). Thus boosting cellular GSH levels prevents barrier impairment (without impacting cell survival, Supplementary information Fig. S4).

### 3.7 Exogenous GSH suppresses occludin tyrosine phosphorylation

**Fig. 7:**
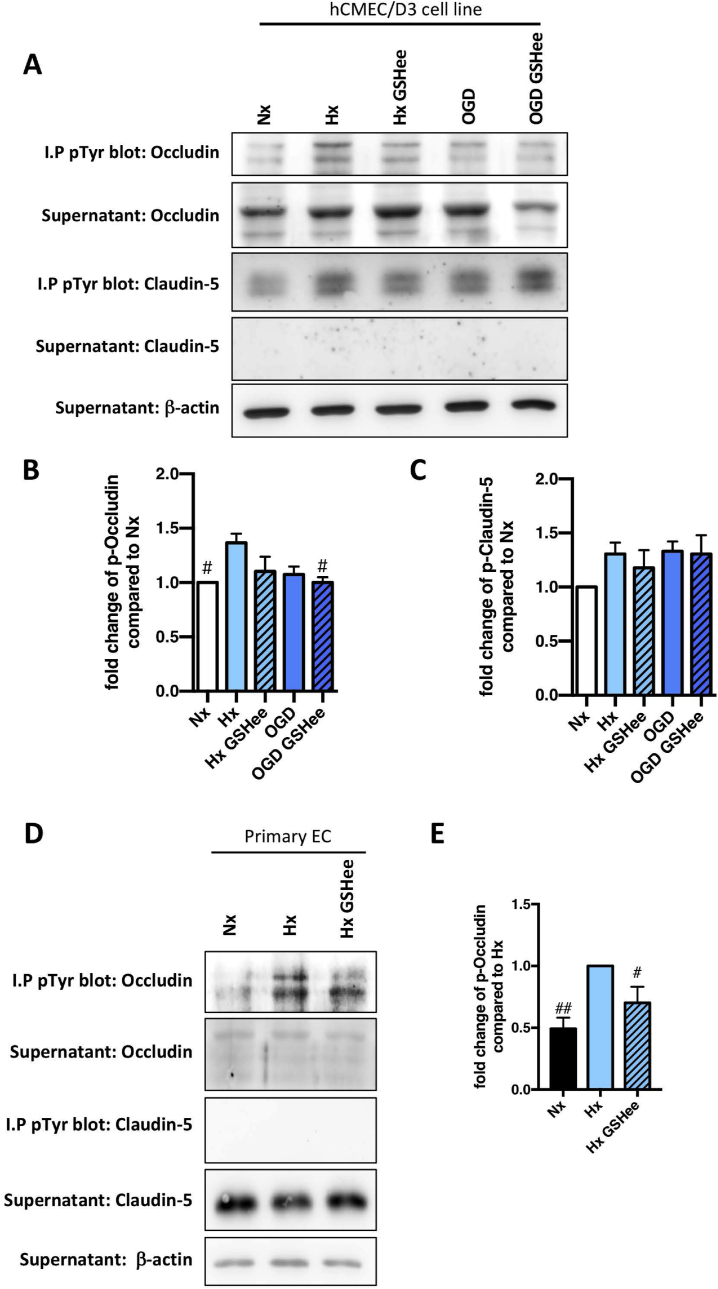
Exogenous GSH suppresses occludin tyrosine phosphorylation. Two different cell models (human brain microvascular EC cell line (hCMEC/D3) and primary EC) were treated with GSHee prior to hypoxic or ischemic exposure for 24h. ***(A)*** Immunoprecipitation (I.P) using phospho-tyrosine conjugated (p-Tyr) beads was performed followed by immunoblot analysis of tight junction proteins in hCMEC/D3. ***(B and C)*** Densitometric quantification of pTyr-occludin (p-Occludin)***(B)*** and pTyr-claudin-5 (p-Claudin-5)***(C)*** was subsequently carried out. ***(D and E)*** Similar modulations were further confirmed in primary EC, shown in the representative immunoblots ***(D)*** and densitometric quantification ***(E)***. I.P pTyr blot shows the pTyr protein level whereas supernatant blot shows the unpulled protein. β-actin as a loading control. ^#^P < 0.05, ^##^P < 0.01; one-way ANOVA compared to Hx baseline. n = 5 in hCMEC/D3; n = 4 in primary EC.

The post-transcriptional modification of tight junction proteins dynamically regulates complex assembly and localization [19,20]. Particularly tyrosine phosphorylation of occludin and claudin-5 C-terminus impacts their interaction with ZO-1 and cytoskeleton, and initiates protein internalization [21,22]. Immunoprecipitation was used to assess claudin-5 and occludin tyrosine phosphorylation (pTyr) status after 24h exposure to hypoxia/OGD in a human brain microvascular endothelial cell line (hCMEC/D3) and primary rat brain EC. As expected increased phosphorylation of both proteins was seen in hypoxic hCMEC/D3. GSHee strongly suppressed hypoxia-induced (Fig. 7A and B) but not OGD-induced (Fig. 7B) occludin tyrosine phosphorylation compared to the normoxic condition, an observation also noted in primary isolated EC (Fig. 7D and E). In contrast, no effect on phosphorylation status of claudin 5 was detected (Fig. 7C).

## 4 Discussion

Multiple studies suggest that stabilizing brain vascular function in patients with neurological disease could arrest or even reverse the course of brain disorders [2]. We are convinced that better understanding of the mechanisms by which perivascular cells support barrier function could provide new insight for future strategies aimed at modulating barrier function. This is the first study to prove the existence and importance of AC-EC metabolic cross-talk under different environmental conditions. We show that constitutive GSH shuttling from AC to EC is strongly increased during injury conditions and suppressing the shuttling significantly reduces AC protective effects and induces barrier dysfunction. Notably, exogenous GSH supplementation also preserved barrier stability in the absence of AC. Thus our data suggests that boosting/elevating GSH levels during disease could improve BBB functionality.

GSH is a key regulator of redox-sensitive transcription factors and stress-sensing pathways that boosts the cellular metabolic systems defence against insult. Metabolomic profiling clearly revealed distinct and differential modulation of AC and EC GSH metabolic pathways. Despite being a high oxidative stress cell type [23], EC had relatively low GSH-related metabolite levels at baseline and seemingly exhausted - or perhaps could not replenish - key GSH metabolites during injury conditions. This progressive GSH metabolism shut down agrees with redox imbalance being a primary cause of EC dysfunction [24,25]. In contrast, AC had a highly activated GSH metabolism during stress conditions supporting observations that this pathway is well maintained under hypoxia and OGD [26,27].

Astrocytes play an important role in antioxidative metabolism and detoxification [5]. AC constitutively secrete GSH under baseline normoxic conditions and are reservoirs of this key anti-oxidant and likely other important metabolites [6]. This characteristic can clearly benefit surrounding cells. Notably, GSH secretion was increased without influencing intracellular levels suggesting activated systems boost its release - although no GSH-specific transporter, exporter or importer, has been identified to date. GSH can however be co-transported with organic anions by different membrane proteins. Our data suggests MRP2 is most likely involved in AC GSH release during injury.

Is GSH itself taken up directly by cells? Interestingly, intravenously injected GSH-coated nanoparticles targeted to EC for drug delivery were observed to successfully accumulate in brain tissue [[28], [29], [30]], but how they enter is not understood. In this study the fact that the isotope is detected in endothelial cells proves that labeled GSH (i.e. of AC origin) is shuttled from AC to EC. Unfortunately, as our isotope labels GSH on sulfur and nitrogen but not specifically on cysteine, whether it can be directly absorbed remains debatable although most evidence does not support this scenario [31]. If indirectly taken up by EC various amino acid importers that transport GSH breakdown products should be taken into consideration. In neurons numerous importers indirectly participate in GSH absorption, including excitatory amino acid transporters (EAAT) 1–3 and Xc^−^ antiporter (XCT) that regulate cysteine and glutamate levels to facilitate GSH synthesis [32,33]. Although we did not investigate this mechanism in detail, it was interesting that XCT mRNA levels (cysteine importer) were also induced in EC during injury conditions (data not shown). Utilizing this route would mean extracellular GSH catabolism is required [34]. Particularly, γ-glutamyl transpeptidase (GGT) might play a key role in releasing glutamate, cysteine and related peptides from AC GSH. Notably GGT has been detected in all BBB compartments including EC [35], pericyte [36] and AC [37]. Since injury conditions induced mRNA levels of AC GGT, as observed in various organs [38,39], we speculate that GGT-mediated GSH breakdown and XCT activation could jointly contribute to GSH recycling and shuttling to the EC compartment. For better insight a more detailed investigation of the precise mechanism is clearly warranted.

Paracrine metabolic processes clearly have a large part to play in regulating BBB stability. Even mild reduction of AC GSH secretion (10–20%) significantly increased barrier permeability implying perivascular metabolic disturbance may be a causative factor in worsening barrier integrity. It is highly probable that to ensure their own survival during harsh situations AC might withdraw their metabolic support leaving vascular 10.13039/501100000780EC to fend for themselves. Such a switch may explain the unexpected negative effects of AC on barrier stability observed during severe conditions [4,18,40]. In this regard GSH supplementation could significantly benefit vascular function. Indeed GSHee (a membrane permeable GSH analog) and NAC (a source of cysteine for GSH generation) administration prevented injury-induced EC permeability as seen in epithelial and hepatocyte studies [41,42]. We noted that GSH treatment prevented occludin tyrosine phosphorylation (pTyr), a modification well known to determine EC tight junction localization, complex stability and integration with regulatory proteins [[43], [44], [45]]. This data also aligns with occludin being a major target for hypoxia induced pTyr by c-Src tyrosine kinases [44,46]. Two different mechanisms may underlie GSH protection; 1) suppression of hypoxia-induced ROS imbalance to prevent the phosphorylation and 2) a non-ROS mediated pathway; perhaps directly activating protein tyrosine phosphatase (PTP) via glutathionylation [47,48]. The mechanisms at work during OGD are even more unclear as pTyr of both occludin and claudin-5 were unchanged by GSH exposure.

Regardless, our data undoubtedly implies that enhancing EC GSH levels could improve redox homeostasis and suppress injury-induced vascular impairment. Although not yet considered for BBB protection, positive effects of boosting GSH has been demonstrated in some neurological-related clinical trials. As GSH is unstable (easily oxidized) and has a very short half-life NAC, a cysteine analog that boosts GSH synthesis, has been the most commonly used enhancer. Intravenous injection of NAC successfully increased brain GSH levels in Parkinson's disease, Gaucher disease and healthy human brain as measured by Tesla magnetic resonance spectroscopy (ClinicalTrials.gov: NCT01427517 and NCT02445651) resulting in general patient improvement [49]. AD patients prescribed NAC over six months also showed significantly improved memory performance [50,51]. However as it is used at relatively high concentrations, NAC has quite a few unwanted side effects [52] and other studies did not observe positive effects (Clinicaltrials.gov NCT00903695 and NCT01320527). As NAC is a precursor, compromised cell function during injury conditions may slow the processes needed for GSH synthesis. Indeed our study supports this view as the membrane permeable GSHee was considerably more effective than NAC. Notably, recent application of GSHee in a stroke MCAo mouse model also reduced infarct size and improved neurological outcome [53]. Thus a stable, cell permeable GSH analog could offer significant clinical advantages.

To conclude, GSH shuttling from AC to 10.13039/501100000780EC supports brain vascular stability during insult and disturbance of this metabolic interaction likely compromizes barrier homeostasis. We thus advocate administration of GSH analogs to boost BBB function and sustain vascular health. Better understanding of the impact of other perivascular-derived metabolites, and paracellular crosstalk, could offer more opportunities to safeguard BBB integrity.

## 5 Material and methods

### 5.1 Reagents

Transwells™ were obtained from Corning (Schiphol, The Netherlands). Lucifer yellow was purchased from Thermo Fisher. Protease inhibitor cocktail Set III from Calbiochem (Merck, Darmstadt, Germany). Oligofectamine™ Reagent and Pierce BCA Protein Assay were from Thermo Fisher Scientific Inc. (Rockford, IL). For Western blotting and immunofluorescence, antibodies directed against occludin, claudin-5, and ZO-1 were purchased from Invitrogen (Basel, Switzerland), β-actin antibody from Sigma–Aldrich (Buchs, Switzerland), and β-catenin antibody from Chemicon (Millipore, Billerica, MA). Anti-phosphotyrosine antibody agarose conjugate, clone 4G10®, was purchased from Milipore. Secondary antibodies for Western blotting and immunofluorescence were obtained from Jackson ImmunoResearch (Suffolk, UK) or Invitrogen. For the glutathione (GSH) and ROS assay, 5,5-Dithio-Bis-(2-Nitrobenzoic acid) (DTNB) and 2′,7′-Dichlorofluorescin diacetate were obtained from Sigma–Aldrich (Buchs, Switzerland). Isotopic labeled ^34^S^15^N-cysteine was designed and synthesized by Dr. T. Sawa (Graduate School of Medical Sciences, Kumamoto University) [54]. γ-glutamyl cysteine ligase catalytic subunit (GCLc) siRNA (ON-TARGET plus SMART pool) and negative control siRNA (ON-TARGET plus non-targeting pool) were purchased from Dharmacon.

### 5.2 Primary cell isolation and cell culture

All cell culture media and reagents were obtained from Gibco® (Life Technologies, Zug, Switzerland) and Sigma-Aldrich. Primary rat astrocytes (AC) were isolated from neonatal pups as described [55] then cultured in DMEM supplemented with 10% FBS and 50 μg/mL gentamycin sulfate on gelatin-coated dishes and used after the first passage. Primary rat brain microvascular endothelial cells (EC) were isolated from 8 to 10 week old male Wistar rats as previously described [56]. EC reached 100% confluence after 7 days culture and were used without passaging. The human cerebral microvascular endothelial cell line HCMEC/D3 was used for immunoprecipitation experiments. HCMEC/D3 were cultivated in Endo GRO™-MV (Millipore) medium containing 5% FBS on collagen-coated culture dishes.

### 5.3 Co-culture methodology

Two different co-culture systems were employed in this study: 1) Contact co-culture model. Primary EC cells were seeded on the upper side of collagen-coated Transwells™ till confluent with AC seeded on the lower side, both at a density of 0.5 × 10^6^ cells/insert. Co-cultures were incubated in DMEM with 10% calf serum (with or without glucose) for 24h. After exposure the cell lysates were collected separately for Time-of-flight mass spectrometer (TOF-MS) analysis. 2) Non-contact co-culture model. As primary cells are very sensitive to trypsin treatment, this model was used to bypass lifting cells after siRNA transfection. AC were cultured at a density of 0.5 × 10^6^ cells/well in 24 well plates whereas EC were cultured as described in the model above. The inserts with EC monolayers were then transferred to the 24-well plates containing transfected AC to initiate the co-culture. These co-cultures were kept in DMEM with 10% calf serum (with or without glucose) for 48h then used for permeability assays.

### 5.4 O_2_ deprivation and ischemic treatment

O_2_ deprivation experiments were carried out in a purpose-built hypoxic glove-box chamber (InVivO_2_ 400, Ruskinn Technologies, Pencoed, UK) maintained at 37 °C with 5% CO_2_. O_2_ concentration was constantly monitored with an internal O_2_ sensor. Cells were exposed to normoxia (Nx, 21% O_2_), hypoxia (Hx, 1% O_2_), oxygen and glucose deprivation (OGD, 1% O_2_ without glucose) and severe OGD (0.2% O_2_ without glucose) for 24h/48h.

### 5.5 Untargeted LC-MS measurements

Non-targeting mass spectrometry measurements and analyses were conducted in cooperation with the Functional Genomics Center Zurich (FGCZ), University of Zurich. Sample preparation and measurements were performed using a nanoACQUITY system coupled to a Synapt G2HD mass spectrometer (Waters Corp., Milford, USA) as previously described [10]. Chromatographic separation of metabolites was performed on a 0.2 μm × 150 mm BEH amide column using a 10 min linear gradient of 90%–50% acetonitrile, 0.5 mM ammonium acetate, pH 9. All analyses were done in negative mode using 1.2 kV capillary voltage, 30 V sampling cone voltage and 3 V extraction cone voltage. The source temperature was set to 100 °C and Nano Flow Gas, i.e. sheet gas flow, was applied.

### 5.6 Data analysis and processing

Waters raw data were first converted to centroid mode and further processed into vendor independent netCDF format using DataBridge (Masslynx, Waters Corp.). Untargeted metabolomics data matrix comprising of accurate mass/retention time information, and ion counts for each sample were calculated using the data processing tool cosmiq [57]. Signal-to-noise ratio (SNR) of mass peak detection was set to 3, SNR for chromatographic peak detection was set to 10 and a *m*/*z* bin size of 0.003 Da was chosen as parameters for cosmiq. For metabolite annotation, the resulting list was first matched to a list of metabolites with known retention time and mass. For additional annotation of unknown metabolites, the list of accurate masses was matched to the KEGG database assuming [M-H]- adducts. Database hits within a mass window of 0.01 Da were considered.

### 5.7 Data normalization strategy

To be able to compare the relative metabolite quantities between the different treatments and cell types, we performed a normalization approach according to the sum of all detected metabolite ion counts as previous [10]. One of the Nx EC samples was randomly chosen as reference and the normalized ion intensity for each metabolite was calculated by dividing the observed ion intensity by the factor Σis/Σir, where Σis is the summed ion intensity for each individual sample and Σir is the summed ion intensity of the reference sample.

### 5.8 Oxidative stress detection and GSH measurement

ROS formation reflecting intracellular oxidative stress was quantified by fluorometric techniques based on 2′,7′-Dichlorofluorescin diacetate (DCF) oxidation according to the manufacturer's instruction (Sigma-Aldrich). GSH measurement was performed using DTNB (5,5′-dithio-bis (2-nitrobenzoic acid)) as published (Bogdanova et al., 2005). Briefly, intracellular GSH levels were measured from the cell lysates harvested in cell lysis buffer (50 mM Tris, 150 mM NaCl, 1% Triton X-100, 1% NP-40) supplemented with protease inhibitor cocktail (Calbiochem, Darmstadt, Germany), 1 mM sodium orthovanadate, 1 mM dithiothreitol, 0.5 mM phenylmethansulfonyl fluoride and 1 mM EDTA. Total protein concentrations were measured using Pierce BCA protein assay and 50 μg was measured in a 96-well format. Extracellular GSH was detected in 100 μL culture media samples immediately after exposure.

### 5.9 Permeability assay

Permeability assays were performed on Transwells™ with confluent primary EC as previously described [18]. Fresh medium containing the fluorescent dye lucifer yellow was added to the upper compartment. At 0, 15, 30, 45 min aliquots were taken from the bottom compartment. Sample fluorescence was measured with a plate reader (FLx800, Biotek Instruments, Winooski, VT). A clearance slope was established from the measurements obtained at different timepoints and used to calculate permeability coefficient values (Pe) (Rist et al., 1997).

### 5.10 Immunostaining

Primary ECs were grown on coverslips coated with collagen IV. After hypoxic and ischemic exposure cells were fixed in 4% paraformaldehyde, permeabilized in 0.1% Triton X-100 in PBS and incubated with occludin (1:100, Invitrogen), claudin-5 (1:100, Invitrogen), β-catenin (1:100, Chemicon). Cell nuclei were counterstained with DAPI (4’,6-Diamidin-2-phenylindol). Pictures were taken using an inverted fluorescence microscope coupled to an 8-bit CCD camera (Axiocam HR, Carl Zeiss) and processed using ImageJ software.

### 5.11 Immunoblotting

Cells were washed with ice-cold PBS and homogenized in cell lysis buffer (50 mM Tris, 150 mM NaCl, 1% Triton X-100, 1% NP-40) supplemented with protease inhibitor cocktail. After measurement with Pierce BCA protein assay 30 μg protein were separated on denaturing SDS-Page and transferred to a nitrocellulose membrane. Membranes were blocked at room temperature in 5% non-fat dried milk or 5% BSA and subsequently incubated overnight at 4 °C in primary antibodies against β-actin (1:5000), occludin (1:500) and claudin-5 (1:300). Membranes were washed with 0.1% Tween-20 in TBS or PBS then incubated with horseradish peroxidase (HRP) conjugated secondary antibody. Band detection was performed and visualized using a luminescent image analyzer LAS-3000 (Fujifilm, Dielsdorf, Switzerland). Blot quantification (using β-actin as loading controls) was performed using ImageJ software (NIH, Bethesda, USA).

### 5.12 Immunoprecipitation

Protein (1 mg) was suspended in I.P buffer (50 mM Tris–HCl pH 7.4, 150 mM EDTA and 0.5% NP-40, 1 mM NaVO_3_, 1 mM PMSF) then 30 μl of anti-phosphotyrosine antibody agarose conjugate (4G10) was added. After 4h incubation at 4 °C in a circular rotator samples were centrifuged (1000 rpm for 1 min) then the supernatant removed and the beads washed 3 times with I.P buffer. Captured proteins were eluted by incubating at 80 °C in 2x Laemmli buffer then run on a 10% SDS–Page gel.

### 5.13 Metabolite extraction

Cell pellets were collected and washed (75 mM Ammonium Carbonate, pH 7.4), then incubated with cold 40:40:20 acetonitrile:methanol:water mixture for 10 min to quench the metabolism. Supernatants were stored at −80 °C.

### 5.14 Measurement of isotopic labeled metabolites

TOF-MS measurements and analyses were followed the procedure of Fuhrer et al. [58]. Analysis was performed on a platform consisting of an Agilent Series 1200 LC pump coupled to a Gerstel MPS3 autosampler and an Agilent 6550 Series Quadrupole Time-of-flight mass spectrometer (Agilent, Santa Clara, CA) equipped with an electrospray source operated in negative mode [58]. Spectra were recorded in profile mode from *m*/*z* 50 to 1000 with a frequency of 1.4 spectra/s for 0.48 min using the highest resolving power (4 GHz HiRes). Source temperature was set to 325 °C.

### 5.15 Data processing and normalization of isotopic labeled metabolites

All data processing and analysis steps were performed with Matlab R2010b (The Mathworks, Natick). More details on data processing, ion annotation, and statistical analysis are shown in the supplementary data. We present the GSH M+3/M ratio based on its intracellular labeled percentage in EC to enable us to largely exclude the influence of passive release.

### 5.16 siRNA transfection

Oligofectamine™ Reagent was mixed with 100 μM siRNA and used to transfect ACs according to the manufacturer's instructions. After transfection, knockdown efficiency was confirmed using DTNB assay.

### 5.17 Statistics

All results are expressed as mean ± SD from a minimum of three independent experiments. Statistical significance using GraphPad Prism 7 software (La Jolla, CA) was assessed by Students T-test or one-way ANOVA for comparison of different time points within a group and two-way ANOVA for comparison between different groups. A P-value below 0.05 was considered significant.

## Declaration of competing interest

The authors declare no competing interests.

## Figures and Tables

**figs1:**