# Supplementary material for: Astrocyte glutathione maintains endothelial barrier stability
Source: Redox Biol. 2020 May 19;34:101576. doi: 10.1016/j.redox.2020.101576 (PMC7267730; doi:10.1016/j.redox.2020.101576)
Supplement: The following are the supplementary data related to this article:Multimedia component 1 [file mmc1.pdf]

Fig. S1

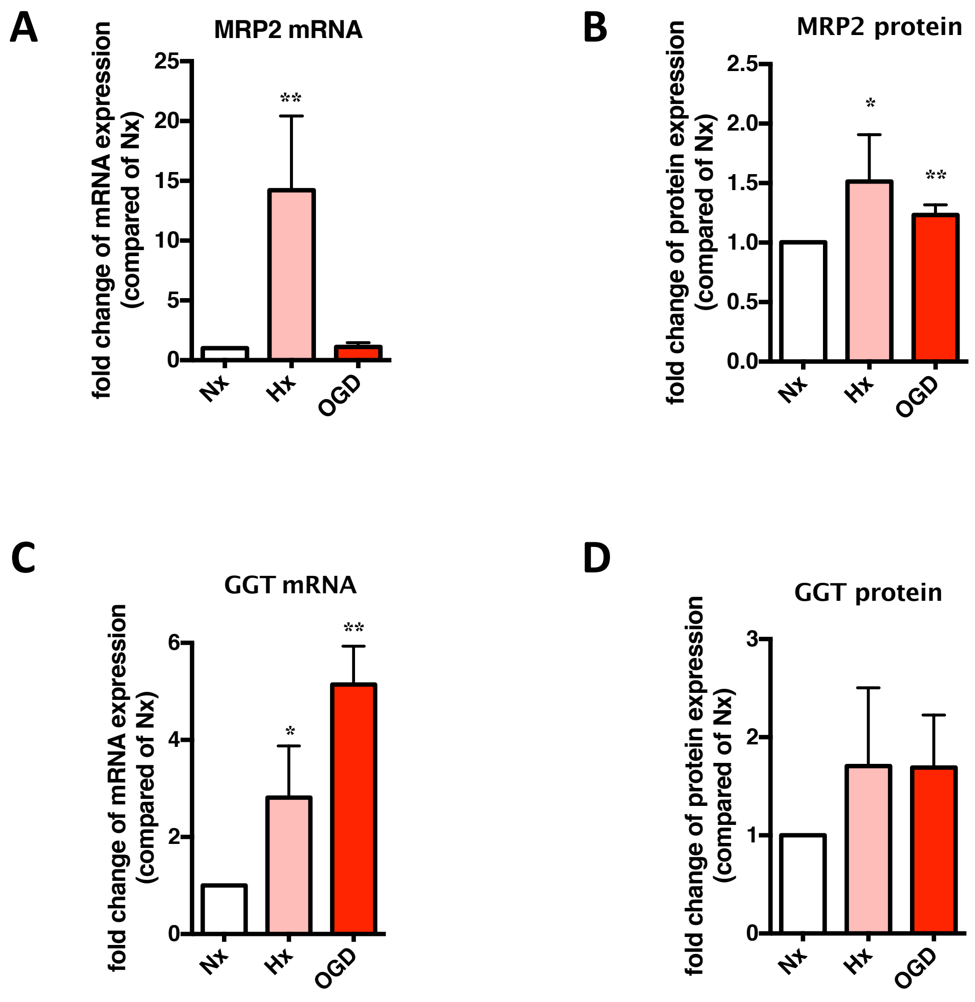

Fig. S1: Injury induces MRP2 transporter and extracellular stabilizer expression

AC lysates were collected after 6h/24h exposure. **a, c** Using quantitative PCR the mRNA level of multidrug resistance-associated protein 2 (MRP2) and γ-glutamyl transferase (GGT) after 6h exposure were assessed. MRP2 and GGT protein expression in AC cell lysates with 24h exposure were assessed by immunoblot. **b, c** Densitometric quantification of MRP2 protein and GGT protein. *P<0.05, **P<0.01, ***P<0.001; One-way ANOVA compared to Nx baseline. Mean± SD. n=4

Fig. S2

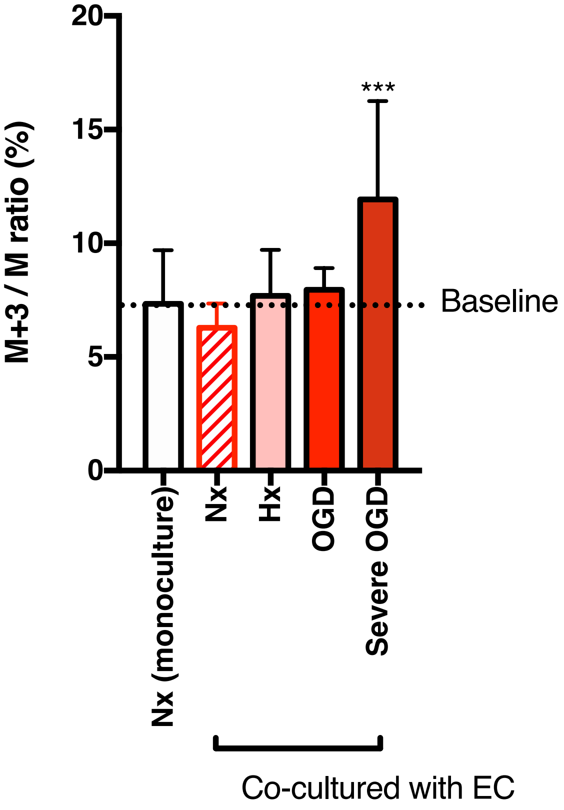

Fig S2: Labeled GSH in AC is diluted after 48h incubation

Isotope-stimulated AC were co-cultured with EC for 24h under different conditions. At experimental end, AC cell lysates were analyzed by TOF-MS. Intracellular $^{34}S^{15}N$-GSH levels in AC were analyzed based on M+3/M ratio. ***$P<0.001$; Student's T-test compared to baseline. Mean $\pm$ SD. n=4

Fig. S3

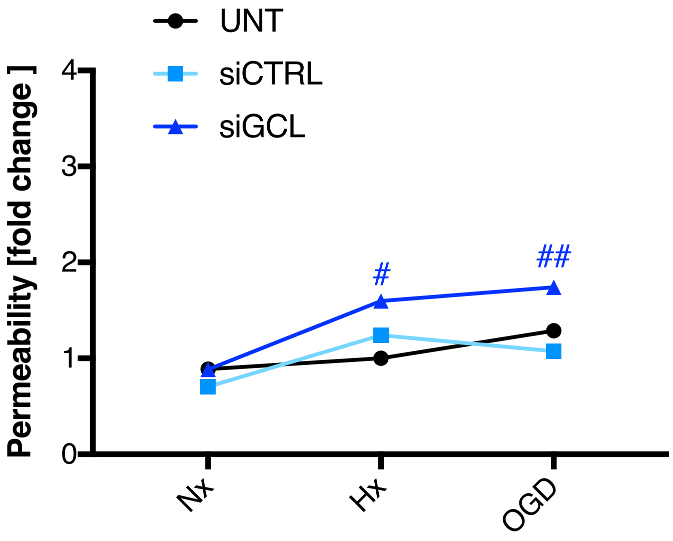

Fig. S3: GSH-deficient AC lose the ability to prevent injury-induced BBB opening

After 48h GCL siRNA transfection, AC were co-cultured with EC and exposed to hypoxia (Hx) and OGD for 48h. At the end of experiment, barrier leakage was measured using lucifer yellow. Fold changes of permeability were compared to Nx UNT. #P<0.05, ##P<0.01; Student's T-test compared to injury control (siCTRL). n=8.

Fig. S4

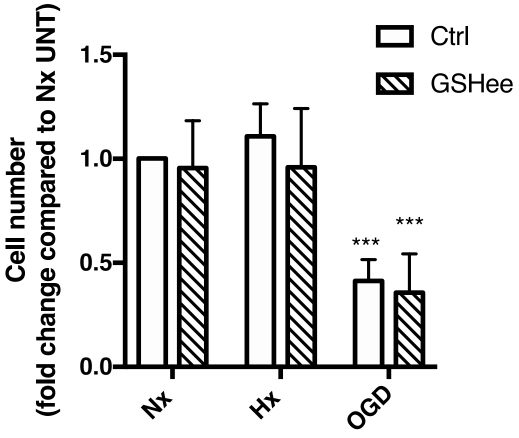

Fig. S4: GSH enhancer does not impact cell survival

hCMEC/D3 were exposed to 48h hypoxia/OGD. Cell nuclei were stained by DAPI and cell counting was performed by automatic cell counter. ***P<0.001; Two-way ANOVA compared to Nx baseline. Mean ± SD. n=3
